# New-Onset Diabetes Presenting as Monoballism Secondary to a Mixed Hyperglycemic Crisis

**DOI:** 10.7759/cureus.2882

**Published:** 2018-06-26

**Authors:** Miguel A Garcia-Grimshaw, Amado Jimenez-Ruiz, Alberto Ornelas-Velazquez, Adrian Luna-Armenta, Francisco A Gutierrez-Manjarrez

**Affiliations:** 1 Internal Medicine/Hospital General De Tijuana, Universidad Autonoma De Baja California, Tijuana, MEX; 2 Neurology, Instituto Nacional de Ciencias Medicas y Nutricion Salvador Zubiran, Ciudad de Mexico, MEX; 3 Internal Medicine, Hospital General De Tijuana, Tijuana, MEX; 4 General Medicine, Universidad Autonoma De Baja California, Tijuana, MEX; 5 Neurology, Hospital General De Tijuana, Tijuana, MEX

**Keywords:** monoballism, hyperglycemic crisis, ballism, hemiballism, movement disorder, hyperkinetic

## Abstract

Monoballism secondary to a mixed hyperglycemic crisis is a rare initial symptom of new-onset diabetes, which commonly affects the elderly and Asian women having inadequate glycemic control. In hyperglycemic states, the elevated serum glucose levels raise the viscosity of the blood reducing cerebral perfusion, decreasing gamma-aminobutyric acid levels, the latter being an inhibitory neurotransmitter of thalamocortical stimuli. In this case, we report a previously healthy 41-year-old male who attended the emergency department because of an abrupt onset movement disorder of the left arm, this being compatible with monoballism. He was diagnosed with a mixed hyperglycemic crisis.

## Introduction

Ballism is a subtype of a hyperkinetic movement disorder. It is considered within the spectrum of chorea as it shares some clinical and pathophysiological similarities. This disorder is described as a sudden onset, arrhythmic, uncontrollable movement of wide amplitude, predominantly in the shoulder and hip and frequently involving the same side of the body. It can also involve only a single limb (monoballism), which is rare, being the upper extremities and the most commonly affected. The lesion typically is localized in the contralateral subthalamic nucleus or in the afferent/efferent pathways of these nuclei. The most common causes of ballism are ischemic or hemorrhagic strokes followed by hyperglycemic crises, the latter being a reversible cause [1].

## Case presentation

A previously healthy 41-year-old male, presented to the emergency department (ED) complaining about involuntary movements of the left arm and abrupt onset that had started 12 hours prior to the admission. He complained of asthenia, adynamia, polyuria, and hyporexia for the last three days. Upon arrival, his blood pressure was 129/82 mmHg with a heart rate of 101 beats per minute; the respiratory rate was 20 breaths per minute and the temperature was 36.4°C. The capillary glucose level was 566 mg/dL. On physical examination, his left arm had a persistent and arrhythmic violent high-amplitude movement, mainly affecting the proximal muscles, which were consistent with monoballism (Video 1). The patient was alert and co-operative. Speech, cranial nerves, strength, muscle stretch reflexes, and cerebellum examination were unremarkable.  

**Video 1:** Abnormal movements of the patient. Monoballism of the left arm.

The patient’s initial blood workup showed a serum sodium of 145 mmol/L (normal range: 135–145) with a corrected sodium of 152 mmol/L for a glucose of 517 mg/dL; potassium 3.7 mmol/L (normal range: 3.6–5), chloride 88 mmol/L (normal range: 98-107), magnesium 0.73 mmol/L (normal range: 0.66-1.85), calcium 2.5 mmol/L (normal range: 2.15-2.5), and serum lactate 1.8 mmol/L. An arterial blood gas analysis showed a moderate metabolic acidosis with a pH of 7.4 and a bicarbonate of 8.7 mmol/L; a calculated osmolarity of 332 mOsm/L (normal range: 285–295) and a high anion gap of 48 mmol/L (normal range: 8-16). Urinalysis was relevant for glycosuria (1,000 mg/dL) and ketonuria (80 mg/dL). These findings were consistent with a mixed hyperglycemic state (ketoacidosis and hyperosmolar state). Complete blood cell count and renal function tests were within the normal range.

**Figure 1:**
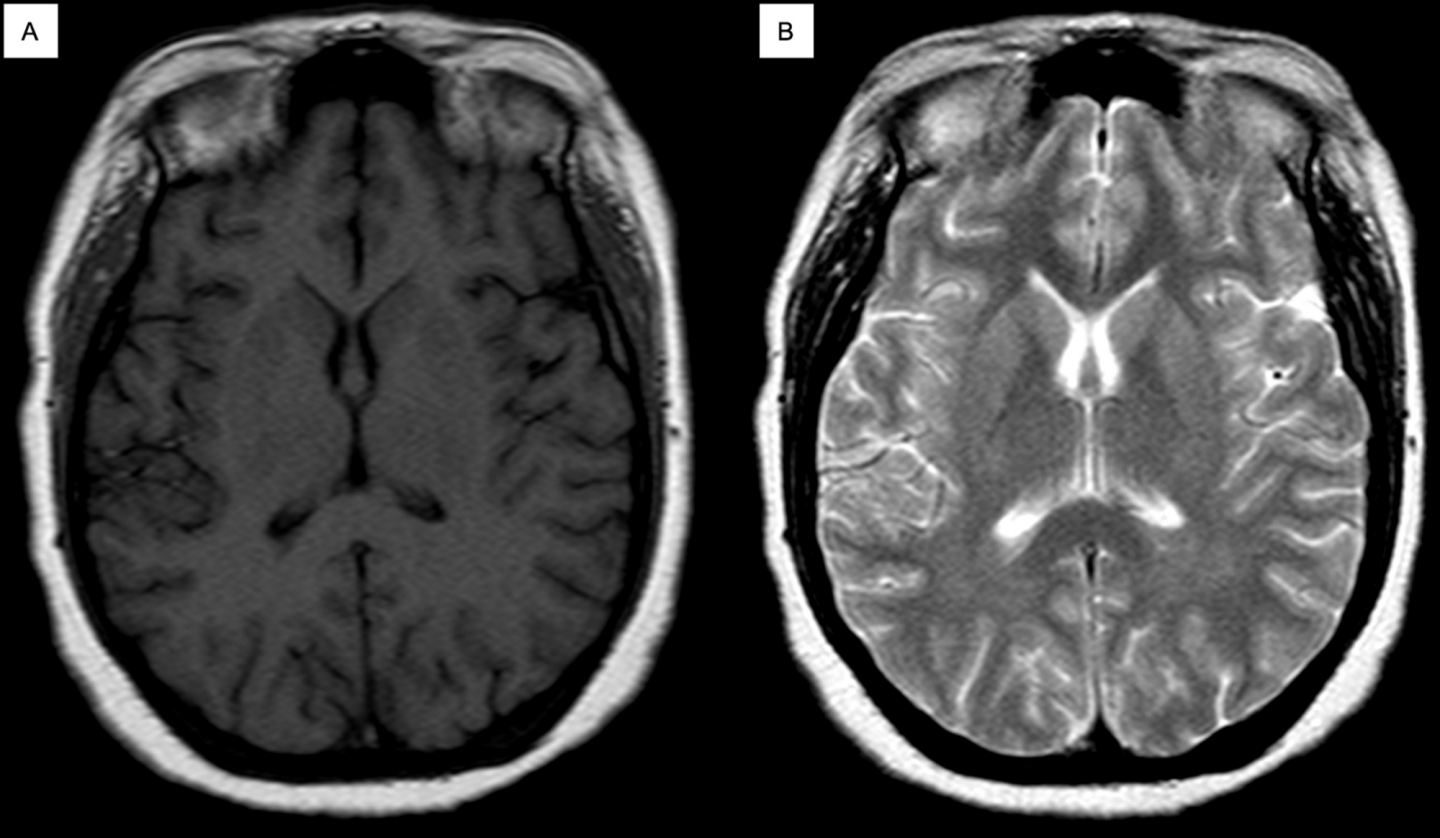
Case MRI images. (A) Axial noncontrast MRI in T1-weighted sequence without lesions in basal ganglia. (B) Axial noncontrast MRI in T2-weighted sequence without lesions in basal ganglia. MRI: magnetic resonance imaging.

The magnetic resonance imaging (MRI) of the brain was normal (Figure 1), without any evidence of ischemia or hemorrhage. In the ED, the patient was treated with normal saline and insulin infusion, which resolved the acute hyperglycemia and the acid base disorder. After resolving the latter, the patient was moved to the internal medicine department. Monoballism resolved 48 hours after correction of the metabolic abnormalities and the patient was discharged four days after admission with insulin as the treatment for his new-onset diabetes.  

## Discussion

The most common cause of ballism is stroke, being present in 4% of these cases. It was followed by nonketotic hyperosmolar hyperglycemic state, the latter being described for the first time in 1960. This movement disorder had an unknown incidence, affecting patients with inadequate glycemic control, and it was frequently reported in the elderly and Asian women [1-3].

There are several theories to explain the appearance of this hyperkinetic movement disorder; one of them is the decrease in cerebral perfusion due to hyperviscosity; another one is the petechial hemorrhages of the striatum; finally, low gamma-aminobutyric acid (GABA) due to reduction in acetoacetate production secondary to brain hypoxia, acetoacetate being the substrate for GABA which functions as an inhibitory neurotransmitter of thalamocortical stimuli [4-6]. 

Typically, the patient presents with acute onset, involuntary, irregular, wide amplitude movements, without a specific pattern involving arms and legs on the same side of the body, in most cases of proximal predominance. Facial involvement is seen in up to 50% of the cases. The intensity of the movements typically increases with intention, improving at rest and disappearing during sleep. The difference between ballismus and chorea according to other authors is that the former one is mainly proximal compared to chorea, which is usually distal [6-7]. 

The differential diagnoses that should be considered are: medication use, specially levodopa and hormone replacement therapy (estrogens); genetic causes like Wilson's disease and Huntington's chorea in young patients; infectious causes such as post-streptococcal Sydenham's chorea, human immunodeficiency virus infection; metabolic causes like hyperthyroidism and lastly antiphospholipid syndrome and carbon monoxide poisoning [4-5]. 

The MRI findings include hyperintensities in the T1-weighted sequence, and more than 50% of the patients may show hyperintensities on the striatum and globus pallidus in T2-weighted sequence with restriction in the diffusion-weighted imaging sequence. These findings are reversible after correction of the underlying metabolic abnormality but may persist up to six years after resolution of the symptoms; spectroscopy may show low N-acetylaspartate/creatine ratio and elevated choline/creatine ratio associated with a peak in lactate concentration [8-9]. A review, which included neuroimaging studies of 120 patients concluded that 18% have lesions in the subthalamic nucleus, 53% have lesions outside the subthalamic nucleus, and 20% have no lesions [7]. The diagnosis is established by physical examination, compatible neuroimaging studies, in association with hyperglycemia, this last one being the key criterion. The treatment must be based upon correcting the hyperosmolar state secondary to hyperglycemia. Usually, resolution of the symptoms occurs within 24-48 hours after glycemic control [6, 10-11].

## Conclusions

Here we report a patient with new-onset diabetes who presented to the ED with monoballism secondary to a mixed hyperglycemic crisis that resolved after achieving glycemic control. It is important to remember this etiology as an infrequent cause of ballism, monoballism being one of the most uncommon initial manifestations of new-onset diabetes, and that the treatment should be oriented towards the correction of the metabolic abnormalities. The other important feature of this case is that the patient had a normal MRI, which can occur in up to 20% of all patients with this hyperkinetic movement disorder. Clinicians should be aware of the underlying metabolic disturbances when approaching a patient with monoballism, as its correct diagnosis and treatment can promptly resolve all neurological signs and symptoms.
